# Automatic Assessment of Keel Bone Damage in Laying Hens at the Slaughter Line

**DOI:** 10.3390/ani11010163

**Published:** 2021-01-12

**Authors:** Lisa Jung, Abozar Nasirahmadi, Jan Schulte-Landwehr, Ute Knierim

**Affiliations:** 1 Farm Animal Behaviour and Husbandry Section, University of Kassel, Nordbahnhofstr. 1a, 37213 Witzenhausen, Germany; uknierim@uni-kassel.de; 2 Agricultural and Biosystems Engineering Section, University of Kassel, Nordbahnhofstr. 1a, 37213 Witzenhausen, Germany; abozar.nasirahmadi@uni-kassel.de; 3 CLK GmbH, Zur Steinkuhle 3, 48341 Altenberge, Germany; schulte-landwehr@clkgmbh.de

**Keywords:** keel bone damage, precision livestock farming, laying hen, health indicators, animal welfare assessment, laying hen health, automated assessment

## Abstract

**Simple Summary:**

Keel bone damage (KBD) is very prevalent in commercial laying hen flocks with a wide range of affected hens/flock. It can cause pain, and affected hens have been found to be less mobile. The assessment of this animal welfare indicator provides important feedback for the farmer about flock health and consequently on the need for interventions. However, the assessment of keel bone damage is time-consuming, and prior training is needed in order to gain reliable results. Optical detection methods can be a means to automatedly score hens at the slaughter line with high sample sizes and in a standardized way. We developed and validated an automatic 3D camera-based detection system. While it generally underestimates the presence of KBD due to the purely visual assessment and technical constraints, it nevertheless shows good accuracy and high correlation of prevalences with those visually determined by a trained human assessor. Therefore, this system opens up opportunities to better monitor and combat a severe animal welfare problem in the long-term.

**Abstract:**

Keel bone damage (KBD) can be found in all commercial laying hen flocks with a wide range of 23% to 69% of hens/flock found to be affected in this study. As KBD may be linked with chronic pain and a decrease in mobility, it is a serious welfare problem. An automatic assessment system at the slaughter line could support the detection of KBD and would have the advantage of being standardized and fast scoring including high sample sizes. A 2MP stereo camera combined with an IDS imaging color camera was used for the automatic assessment. A trained human assessor visually scored KBD in defeathered hens during the slaughter process and compared results with further human assessors and automatic recording. In a first step, an algorithm was developed on the basis of assessments of keel status of 2287 hens of different genetics with varying degrees of KBD. In two optimization steps, performance data were calculated, and flock prevalences were determined, which were compared between the assessor and the automatic system. The proposed technique finally reached a sensitivity of 0.95, specificity of 0.77, accuracy of 0.86 and precision of 0.81. In the last optimization step, the automatic system scored on average about 10.5% points lower KBD prevalences than the human assessor. However, a proposed change of scoring system (setting the limit for KBD at 0.5 cm deviation from the straight line) would lower this deviation. We conclude that the developed automatic scoring technique is a reliable and potentially valuable tool for the assessment of KBD.

## 1. Introduction

The keel, also called the breast bone, is the biggest bone in laying hens. It runs axially along the midline and extends outward, perpendicular to the plane of the ribs. Keel bone damage (KBD) can occur in non-cage systems in high prevalence. It has been found by visual inspection and palpation of hens in 100% of flocks with a wide range of 3–97% hens/flock affected [1,2,3,4]. KBD can consist of either deviations or fractures or both, but without dissection of the bone or other methods like histology or radiology, it is not possible to reliably distinguish mere deviations from fractures. In fact, results from Scholz et al. [5] suggest that most deviations are accompanied or caused by fractures. A correlation of distinct deviations and fractures could also be shown, e.g., by Fleming et al. [6] and Saraiva [7]. A host of factors contributes to its occurrence related to management and rearing, nutrition and housing [8]. Keel bone fractures are a serious welfare problem that involves pain [9,10] and impairment of mobility [2,11,12]. However, laying hen farmers are often not informed about the keel bone status in their flock and therefore are unaware of this welfare problem.

KBD can be assessed on-farm by palpation and visual inspection, but the handling and assessment of a sufficient number of animals in order to achieve a reliable estimation of the flock prevalence is an obstacle in the implementation of routine welfare monitoring on-farm using animal-based indicators (e.g., [13] for cattle, [14] for pigs). Nevertheless, increasingly, retailers and, in some countries like Germany, welfare law [15] require animal-based welfare monitoring concerning a number of potential welfare problems. In the search for less time-consuming assessment methods, the bottleneck slaughterhouse comes into particular focus, as at this point a high number of animals or even the whole flock can easily be inspected. The results provide retrospective information about on-farm welfare, as long as indicators are not largely influenced by catching and transport, which could possibly cause further keel bone fractures. Furthermore, assessments can be automated, which has the further potential advantage of better standardization and consequently increased reliability of assessments. The latter is important because repeatability of KBD assessment between different assessors is demanding to achieve and requires intensive training [16,17].

For an automated assessment, image processing techniques can be applied. They are a cheap, non-contact and cost-effective tool, and have already been used to monitor diverse livestock health and behavior measures (reviewed by [18,19,20]). Automatic, image-based assessment systems are routinely applied to, e.g., the evaluation of foot pad lesions in broilers and turkeys [21,22,23]. Even though some of the keel bone fractures, which are not associated with deviations from the straight line or clearly visible callus formation, are not detectable by visual inspection (and only to a limited degree by palpation), a major part of KBD, including deviations and thereby fractures, is clearly visible and therefore a good candidate for image processing.

The current research project aimed to develop and validate a measurement system for an automated image-based recording of KBD in laying hens at the slaughterhouse in order to allow a time-efficient and reliable assessment and thereby to contribute to an improvement of laying hen welfare.

## 2. Animals, Materials and Methods

### 2.1. Slaughterhouse and Animals

Development of the automated system took place in the years 2017 to 2019, with validation at a poultry slaughterhouse in Germany, involving laying hens of different genetics (e.g., Lohmann Brown, Lohmann Selected Leghorn, Novogen) at the end of lay. The commercial slaughter process comprised manual unloading from the transport crates and shackling, electrical stunning in a water bath, bleeding to death and afterward plucking by a roller fitted with rubber studs. Visual KBD assessments were always carried out after defeathering.

### 2.2. KBD Assessment by Human Assessors

KBD assessment was carried out by one trained person via visual inspection (score 0: intact, no visible callus or dislocation; no lateral, dorsal or ventral deviation from straight axis ≥ 0.2 cm; score 1: damaged, visible callus; dislocation or deviation from straight axis > 0.2 cm). At the slaughter line, the assessor was standing at around 30 cm distance from the carcasses. Chain speed slightly differed but was not faster than one carcass/two seconds. For the assessor, it was possible to follow the carcasses for five more seconds.

Inter-assessor reliability was tested in ten different pairs of one trained person and an experienced or inexperienced further assessor. Five persons were untrained and nine trained. The assessor pairs simultaneously scored 46 to 400 randomly selected hens (a total of 2273 hens) at the slaughter line as well as outside the slaughter line during the normal slaughter process.

### 2.3. Camera Vision System

**Figure 1:**
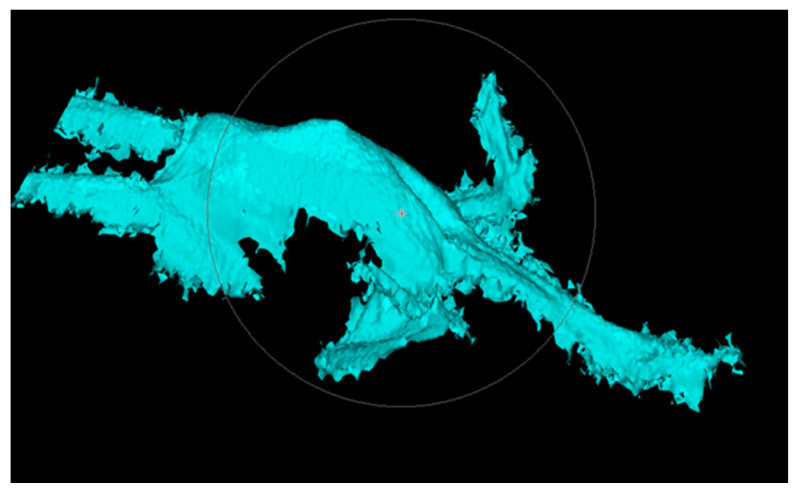
Example of a blue pattern on the carcass.

A 2MP stereo camera with an active pattern projector to enhance the 3D reconstruction and a 5MP color cam were used in combination for the automatic assessment. The stereo camera included two monochrome cameras with a resolution of 1280 × 1024 pixels and an 8mm lens and working range of 480–1000 mm. The 3D image was improved by the projection of a blue pattern onto the carcass (Figure 1). The cameras were fitted in a stainless-steel housing with an IP69 similar protection class. The image recording was triggered by an infrared light barrier when there was a hook in front of the camera. At the focal point, which was 650 mm for this camera, the Z accuracy was set to 0.715 mm. The picture of the carcass was immediately processed after the picture was taken. The 3D reconstruction was performed on a NVidia Geforce GTX 1080 TI graphics card due to the high belt speed. The evaluation computer was also equipped with an Intel i7–6700 processor and 8 GB RAM. The algorithm for the 3D reconstruction was provided by the camera manufacturer and can be modified using various parameters. For the evaluation of the 3D data, the image processing library Halcon of the company MVTec was used.

### 2.4. Development and Validation of the Automated System

**Figure 2:**
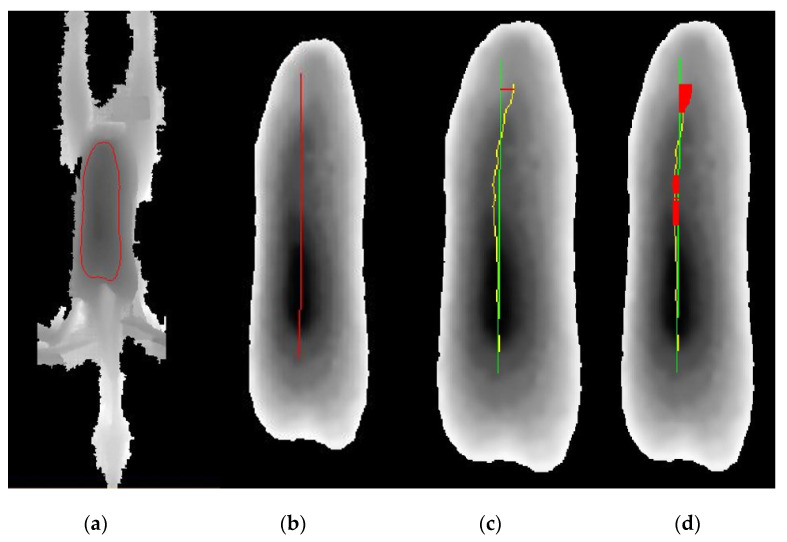
Image examples used for the automatic scoring of keel bones of laying hens at the slaughter line. Image (**a**) shows the keel area. Image (**b**) the straight main axis and (**c**) the deviation (yellow line). Image (**d**) shows in red the detected deviation.

To identify the keel, the breast area was first determined on the Z-image (Figure 2a). In this elliptical region, a line was drawn by the user, presenting the main axis by calculating the center region (Figure 2b). The actual course of the keel was extracted using height information. The maximum and mean horizontal distances between the ideal and actual keel line were calculated (Figure 2c). The keel was classified as damaged if the mean distance from the ideal line was ≥0.2 cm (Figure 2d). Algorithm development was carried out in the following stages:

Initial phase: Altogether, 2287 hens of different genetics with varying degrees of KBD from more than 12 slaughter batches were temporarily taken from the slaughter line on two different days and hung in a self-made scaffold. KBD was assessed by one trained person by visual inspection, standing directly in front of the carcass. In case of doubt, a ruler was used to measure the deviation. After the assessment by the observer, the carcass was recorded with the camera system. Based on these assessments, the initial algorithm was developed.

Learning phase: The camera system was installed at the slaughter line. Five slaughter batches were assessed by the trained assessor by visual inspection and the automated system. Resulting prevalences of KBD were compared. In total, 2170 hens were assessed by the assessor and 25,748 by the camera during this step. Based on these results, an optimization of the camera and algorithm was carried out.

Optimization step 1: Performance of the system in comparison to the visual scoring by the trained assessor was evaluated using the criteria sensitivity, specificity, accuracy and precision. This was done in two optimization steps on 189 and 60 marked carcasses with differing damage levels. In addition, KBD prevalences of six batches were assessed by a human assessor and an automated system. Moreover, algorithm optimization was based on deviating assessments between the human assessor and the automated system from a scoring of a further 300 carcasses.

Optimization step 2: Again, 93 and 79 marked carcasses were scored and prevalences of four slaughter batches determined. During the last batch, the assessor used a modified threshold for keel bone damage: deviation from straight axis of ≥0.5 cm (in contrast to ≥0.2 cm).

In total, KBD prevalences from 15 batches for the comparison of the prevalence ascertained by assessor and automated system, and individual assessments of 421 birds out of four different batches for calculation of performance criteria (sensitivity, specificity, accuracy and precision), were included in the validation.

### 2.5. Statistics

Inter-assessor reliability was evaluated using the prevalence-adjusted, bias-adjusted Kappa (PABAK): 

PABAK = ((k × p) − 1)/(k − 1)

with k = number of categories, p = proportion of matchings between assessors.

Results were interpreted in line with Landis and Koch [24] as follows:

≤0.20: poor/slight; 0.21–0.40: fair; 0.41–0.60: moderate; 0.61–0.80: substantial; 0.81–1.00: almost perfect.

Performance criteria were calculated as follows:

Sensitivity = *TP*/(*TP + FN*)

Specificity = *TN*/(*TN + FP*)

Accuracy = (*TP* + *TN*)/(*FP* + *FN* + *TP* + *TN*)

Precision = *TP*/(*FP* + *TP*),

with *TP* = true positives (damaged keels recorded as damaged), *TN* = true negatives (non-damaged keels recorded as intact), *FP* = false positives (non-damaged keels recorded as damaged keels), *FN* = false negatives (damaged keels recorded as non-damaged keels). The PABAK was also calculated regarding the agreement between human assessor and automated recording.

These analyses were done in Excel 2016. In addition, the relation between KBD prevalences determined by the automated system or human assessor was investigated by Pearson correlation analysis (SPSS Version 27).

## 3. Results

### 3.1. KBD Assessment by Human Assessors

Inter-assessor reliability was higher with trained compared to untrained assessors (mean PABAK: 0.76 versus 0.72), but was substantial in all cases except one (with a moderate result) and almost perfect in four cases (Table 1). More detailed data for inter assessor reliability are provided in the Supplementary Materials (Table S1).

### 3.2. Validation of the System

Performance of the system improved during optimization. It finally reached performance levels of above 80% for all criteria except for specificity with 77% (Table 2 and Table 3). More detailed data of performance assessments are provided in the Supplementary Materials (Table S2).

KBD prevalences ascertained by the human assessor in the 15 flocks during learning and optimization phases ranged between 23.0% and 68.9% (mean: 42.1%, median: 39.2%). After the modification of score 1 in the last assessment round to: deviation from straight axis of ≥0.5 cm (in contrast to ≥0.2 cm)the prevalence was reduced by 11.4% points to a minimum prevalence of 18.8%. At the same time, the lowest difference between automated and human assessment of 1.5% points was achieved here. Otherwise, differences ranged between a maximum of 32.5% points during the learning phase and a minimum of 1.5% points in optimization step 2 (mean: 7.9% points in optimization step 2; Table 4). Overall phases prevalences ascertained by human and automated assessment showed a substantial correlation (r = 0.687, *p* = 0.003, *n* = 16, assessment round 15b not included)

## 4. Discussion

The visual scoring of animal-based measures by a human assessor is an important method in livestock welfare assessments. However, it is time-consuming and labor-intensive [18] and requires a minimum of training. To our knowledge, this project was the first attempt to develop and evaluate a system for the automatic detection of KBD at the slaughterhouse.

In the present project, the keel status was purely visually evaluated, which leads to an underestimation of the damage, since smaller deviations, dislocations and callus deposits can only be detected by palpation or other methods such as radiography. In addition, we found that the camera further underestimates the occurrence of KBD. In part, the number of damaged keels that were not correctly recognized by the camera system might have been due to the movement of the carcass during imaging. However, the exposure time of the camera was very low (<2 ms), so problems due to motion blur are unlikely. By using a 3D camera with a higher resolution, accuracy could be improved, but this would more than double the costs of the system, possibly rendering it less attractive for commercial application. Another option may be an increase of framerate from the current one image for each shackle to more images of each chicken from different perspectives, integrating the different 3D models into one big model with more information and a higher resolution. However, through optimization of the algorithm, we were able to already reach a high sensitivity of 95% and a specificity that approached 80%. The agreement between human observer and automatic system was also substantial when expressed as PABAK and comparable to agreements between different human observers. In addition, the substantial correlation between the differently achieved prevalences by automated system and human assessor proves that the farmers will still receive a fair reflection of the relative magnitude of the welfare problem in their flock. The general underestimation of KBD cases is a little problematic at the currently rather high prevalence levels in practice but should be considered in the interpretation of the results, especially when it should come to setting up target values. In a further validation step, both for the automated system and the human assessment by visual inspection and palpation, additional scoring results should be compared to true KBD ascertained by the scoring of dissected bones, radiographs or other kinds of bone visualizations. Moreover, possible influences of catching and transport on KBD should be determined.

A change of the scoring scheme to the definition of a damaged keel with deviations and dislocations ≥0.5 cm led to a considerably better agreement between assessor and camera as well as to an improvement of sensitivity, specificity, accuracy and precision. At the same time, it will likely also improve inter-assessor reliability further, although predominantly, substantial to almost perfect agreement was already reached. Regarding the inexperienced assessors, these results were unexpected but may be due to the merely visual assessment being easier than the assessment via palpation. In case of a system failure at the slaughterhouse, this and the higher threshold for scoring a KBD would make it easier for slaughterhouse personnel to reliably determine KBD prevalences by visual inspection. Therefore, we recommend changing the definition of visually assessed KBD accordingly and setting a deviation from the straight line of the keel bone of 0.5 cm as the limit for the scoring of KBD (except if callus material is clearly visible). This, furthermore, corresponds to possible discussions about the severity of the welfare problem if the keel bone shows only deviations and no fractures. Presently, no information about the relation between keel bone deviations and pain is available. However, a more severe deviation is more likely associated with a fracture. Scholz et al. [5] histologically found indications of fractures (callus material or new bone tissue) in 51% of macroscopically assessed keel bones with slight deformations, in 80% of keel bones with moderate deviations and in all keel bones with severe deviations. Thus, deformities of ≥0.5 cm are more likely associated with fractures and therefore with pain. However, more research is needed to gain a better understanding of the types and implications of damage that is represented by only small deviations from the straight line of up to 0.5 cm. More investigation would also be needed on the kinds and levels of fractures that cannot be detected by the automated system as well as by palpation technique. This includes fractures of the tip of the keel that are also difficult to score reliably by human assessors [25]. Here, open questions further relate to causes and welfare effects of these fractures.

The proposed image processing technique in this study can also be improved in a fully automated way by using robust machine learning techniques.

However, the developed system is already able to correctly identify KBD on a satisfactory level. It has the advantage that high sample sizes can easily be achieved and that it opens up the possibility to monitor the development of a severe welfare problem that currently does not receive enough attention in practice.

The disadvantage of retrospective monitoring at the slaughterhouse is that it does not allow an immediate intervention in order to improve the welfare of the individual animal [26]. However, KBD is usually affected by long-term causes such as generally fragile bones, unfavorable housing equipment like metal perches, narrow corridors in aviaries, poor lighting conditions and high laying performance, which cannot be remedied with a simple and quick countermeasure. Automatic recording would help the laying hen farmers gain first knowledge about KBD prevalence in their flocks and thus determine the need for long-term changes. Besides, it would also be an easy method to gain information about the development of KBD in general over years and could support investigations about KBD. Therefore, it could be a highly valuable instrument for the monitoring of hen welfare and provide farmers with important information for their management decisions as well as researchers for future epidemiological welfare studies.

## 5. Conclusions

The successful development of an automatic camera-based detection technique allows the reliable recording of keel bone damage (KBD) at the slaughter line. The system generally underestimates the presence of KBD due to the purely visual assessment and technical constraints. Nevertheless, it shows good accuracy and a substantial correlation of prevalences with those visually determined by a trained human assessor. Considering the current mostly high KBD prevalences in commercial laying hen flocks and the general lack of information of farmers about the magnitude of the problem on their individual farms, this system opens up opportunities to better monitor and combat a severe animal welfare problem in the long term.

## Figures and Tables

**Table 1:** Results of inter-assessor reliability tests (prevalence-adjusted, bias-adjusted kappa: PABAK) for the assessment of keel bone damage in laying hen carcasses by visual inspection between ten different pairs of one trained person and an experienced or inexperienced further assessor.

Year	N of Hens	PABAK	Experience ^1^	Pair No.
2017				
A	110	0.78	yes	1
B	110	0.78	yes	2
C	100	0.90	yes	3
D	157	0.81	yes	3
2018				
E	76	0.82	no	4
F	90	0.73	yes	3
G	90	0.76	no	5
H	150	0.75	yes	6
I	400	0.65	yes	3
J	400	0.78	yes	3
K	400	0.67	yes	7
2019				
L	76	0.82	no	8
M	68	0.62	no	9
N	46	0.57	no	10

^1^ Experience of the further assessor: yes = person who received training and feedback in two or more sessions with scoring of at least 150 hens, no = person who had never scored KBD before.

**Table 2:** Number of true positives, false positives, true negatives and false negatives for four performance assessments.

Assessments	True Positive	False Positive	True Negative	False Negative	N Total
Opt1–1	32	26	50	81	189
Opt1–2	9	3	28	20	60
Opt2–3	15	8	41	29	93
Opt2–4	23	5	34	17	79
Opt2–5 ^1^	38	9	30	2	79

Opt1, Opt2 = Optimization step 1 or 2; ^1^ assessment of the same carcasses as in No. 2–4, but with further optimized algorithm.

**Table 3:** Sensitivity, specificity, accuracy and precision of the automatic keel bone damage detection system installed at the slaughter line.

Assessments	Sensitivity	Specificity	Accuracy	Precision	Prevalence ^1^	True Prevalence ^2^	PABAK	N
Opt1–1	0.28	0.66	0.43	0.55	30.7	59.8	−0.13	189
Opt1–2	0.31	0.90	0.62	0.75	20.0	48.3	0.23	60
Opt2–3	0.34	0.84	0.60	0.65	24,7	47,3	0.20	93
Opt2–4	0.58	0.87	0.72	0.82	35.4	50.6	0.44	79
Opt2–5 ^3^	0.95	0.77	0.86	0.81	38.3	50.6	0.72	79

Opt1, Opt2 = Optimization step 1 or 2; ^1^ determined by automated system; ^2^ determined by trained person via visual inspection; ^3^ assessment of the same carcasses as in No. 4, but with further optimized algorithm.

**Table 4:** Prevalence (%) of keel bone damage in laying hens at the slaughter line assessed by a trained person and an automated camera system and differences between the determined prevalences (% points).

	Assessments	Batch Size	Assessor		Camera		
			N	Prevalence %	N ^1^	Prevalence %	Difference (% Points)
Learning phase	1	15,815	481	39.1	13,315	21.5	17.6
	2	1044	393	38.3	824	41.7	3.4
	3	1780	538	46.7	1426	34.5	12.2
	4	9235	210	55.2	8436	22.7	32.5
	5	2732	548	40.0	1747	21.2	19.0
Opitmization step 1	6	906	665	39.2	762	31.6	7.6
	7	10,510	314	29.3	8985	25.9	3.4
	8	1724	1247	54.1	1411	32.5	21.6
	9	7105	2344	29.0	6241	19.0	10.0
	10a	15,249	1307	68.7	12,845	45.7	23.0
	10b ^2^	15,249	1187	68.9	12,845	45.7	23.2
	11	3091	392	43.6	2663	46.8	3.2
Optimization step 2	12	275	253	32.4	251	24.3	8.1
	13	745	241	35.3	665	21.8	13.5
	14	4255	369	23.0	3784	19.5	3.5
	15a	4516	556	30.2	4103	17.3	12.9
	15b	4516	414	18.8 ^3^	4103	17.3	1.5

^1^ Hens hanging only with one foot or askew in the shackles could not be evaluated by the camera, ^2^ two different recordings on the same batch, ^3^ the last assessment was conducted using a modified threshold for keel bone damage by the assessor: deviation from straight axis of ≥0.5 cm (in contrast to ≥0.2 cm).

## Data Availability

The data presented in this study are openly available in [repository name e.g., FigShare] at [doi], reference number [reference number].

## Supplementary Materials

The following are available online at https://www.mdpi.com/2076-2615/11/1/163/s1, Table S1: Data of inter-observer reliability tests of visually assessed keel bone damage at the slaughter line, Table S2: Data of the assessment of keel bone damage by the automated camera system for performance calculations
